# Decolonizing Epidemiological Research: A Critical Perspective

**DOI:** 10.1055/s-0043-1769088

**Published:** 2023-06-07

**Authors:** Yusuff Adebayo Adebisi

**Affiliations:** 1 Nuffield Department of Population Health, University of Oxford, Oxford, United Kingdom

**Keywords:** decolonization, epidemiology, health equity, power dynamic, inclusivity

## Abstract

Decolonizing epidemiological research is a crucial endeavor. Historically, colonial and imperialistic ideologies have pervaded epidemiology, leading to an emphasis on Western perspectives and the neglect of indigenous and other marginalized communities' needs and experiences. To effectively address health disparities and promote justice and equality, acknowledging and addressing these power imbalances are imperative. In this article, I highlight the need of decolonizing epidemiological research and make recommendations. These include increasing the representation of researchers from underrepresented communities, ensuring that epidemiological research is contextually relevant and responsive to the experiences of these communities, and collaborating with policymakers and advocacy groups to inform policies and practices that benefit all populations. Moreover, I underscore the importance of recognizing and valuing the knowledge and skills of marginalized populations, and integrating traditional knowledge—the distinct, culturally specific understanding unique to a particular group—into research efforts. I also emphasize the need of capacity building and equitable research collaborations and authorship as well as epidemiological journal editorship. Decolonizing epidemiology research is a continual process that requires continuing discourse, collaboration, and education.

## Introduction

Decolonizing epidemiological research is an essential aspect of promoting social justice and health equity on a global scale. In recent times, the need to advance health equity is even more pertinent, and epidemiological research is key to understanding the distribution and determinants of health and disease across populations. 1 This reifies that epidemiological research perpetuating any form of systemic racism or imperialistic ideologies adversely affect our scientific understanding of disease prevention and control. Like many other fields, epidemiology has a long history of being shaped by colonial and imperialistic ideologies, 2 3 and as a result, it has often been perceived to further the agendas of the dominant power structures. The practice of ignoring the historical context of colonialism, racism, and other systems of oppression in epidemiological research is unfortunately all too common. 3 4 5 6 7 Global public health experts have also long recognized that racism perpetuates health disparities. 8 9 10 11 To decolonize epidemiological research, it is crucial to acknowledge and address these historical and ongoing power imbalances. The aim of this article is to provide an overview of the issue of decolonizing epidemiological research methods and offer concrete suggestions for how to address it. It also aimed to engage and educate readers on the importance of this issue and encourage them to act in support of decolonization efforts.

## What Is “Decolonizing” in the Context of Epidemiological Research?

There is not a single universally accepted definition of decolonizing epidemiological research, as the concept of decolonization can be interpreted and approached in various ways depending on the context. 3 6 7 In general terms, decolonizing epidemiological research involves acknowledging and addressing the historical and ongoing influences of colonialism and imperialism in shaping the field of epidemiology, as well as identifying and challenging the biases, assumptions, and power imbalances that have resulted from these influences. This process aims to promote more inclusive, equitable, and culturally sensitive research practices that respect and involve the perspectives and needs of marginalized and underserved communities. Moreover, it seeks to ensure that the benefits of research are equitably distributed and that the research process itself does not perpetuate or exacerbate existing health disparities. A critical aspect of decolonizing epidemiological research is encouraging the inclusion of diverse voices and perspectives in the development of research questions, methodologies, and the interpretation and dissemination of results. In this article, the concept of decolonizing epidemiological research would be viewed from these lenses.

## The Issues

One of the key ways in which colonial and imperialistic ideologies have shaped epidemiology is through the way that research has been conducted. Many early epidemiological studies were conducted by Western researchers in colonized countries, often without the informed consent or participation of the local communities. One example of an unethical epidemiological study conducted in a colonized country is the longitudinal Tuskegee Syphilis Study, which was conducted in the United States from 1932 to 1972. 3 12 13 14 While the Tuskegee Syphilis Study is not an example of Western researchers conducting studies in colonized countries, it remains a significant example of unethical research practices and exploitation of minorities populations, a product of unfortunate lesson learned from colonization. The Tuskegee study was conducted on African American men who were living in poverty and lacked access to healthcare, and they were not informed of their diagnosis or offered treatment. This unethical research practice reflects the power dynamic between the researchers, who were predominantly white, and the participants, who were predominantly African American and from a marginalized community. This reflects the history of racism and discrimination against African Americans in the United States, which has its roots in colonialism and slavery. 15 16

The Guatemala Syphilis Experiment, a nonconsensual human experimentation study with a mix of observational and interventional components, was another notorious human subject research project conducted between 1946 and 1948. 17 18 19 American researchers, led by Dr. John C. Cutler, collaborated with Guatemalan health officials to carry out the study, which aimed to investigate the effectiveness of penicillin in preventing and treating syphilis and other sexually transmitted infections. The experiment involved intentionally exposing vulnerable populations, including Guatemalan soldiers, prisoners, and mental health patients, to syphilis, gonorrhea, and chancroid without their informed consent. 18 The participants were not adequately informed about the risks and potential consequences of the study. 19 Many of those who were infected did not receive proper treatment, even when it was available. 17 18 19 The Guatemala Syphilis Experiment is considered one of the most egregious examples of unethical medical research in history. 17 18 It highlights the issues of power imbalances and the exploitation of vulnerable populations by Western researchers in colonized or developing countries.

Over the past two decades, researchers in colonized countries have advocated for an indigenous approach to epidemiology that incorporates local knowledge, community-based participatory research, and indigenous research methodologies, while enhancing capacity by training more indigenous epidemiologists and supporting indigenous self-determination. 20 21 However, this approach has yet to receive sufficient attention. To understand how geopolitics perpetuates inequities and how integrating local knowledge can help reduce such inequities, we must also address the disciplinary preference for quantitative epidemiological research. 10 This preference reinforces the belief that quantitative research is more rigorous and authoritative than qualitative research, despite the potential of qualitative approaches to provide valuable insights into the social, cultural, and contextual factors that contribute to health disparities. 22 This preference for quantitative research is evident in the default thinking that epidemiological research primarily entails quantitative research, while qualitative research is often underappreciated. This underappreciation of qualitative research ignores its potential to offer valuable insights into health disparities and social determinants of health. 22

An example of class-based oppression in epidemiological research can be seen in studies conducted on environmental health disparities. 23 24 25 26 Low-income and working-class communities, often with a high proportion of racial and ethnic minorities, are disproportionately exposed to environmental hazards such as air pollution, contaminated water, and toxic waste. 27 These communities are often located near industrial facilities, landfills, or major highways, which contribute to poor air quality and other health risks. If epidemiological research fails to account for these social determinants of health, it may overlook or underestimate the effects of environmental hazards on the health outcomes of individuals in these communities. 3 This lack of attention to class-based disparities can result in inadequate policy responses and interventions, further exacerbating health inequities. In addition, class-based oppression in epidemiological research can manifest in the exclusion of low-income communities from decision-making processes, study design, or dissemination of research findings. This exclusion can lead to a lack of culturally sensitive and contextually appropriate interventions, perpetuating the cycle of health disparities and social injustice.

Furthermore, big data has the potential to revolutionize epidemiological research and practice, 28 but it is important to recognize and address the ways in which power imbalances and colonial and imperialist ideologies can shape the use of big data. For instance, the coronavirus disease 2019 (COVID-19) pandemic has highlighted the importance of big data in global health research and practice, 29 but it has also drawn attention to the unequal distribution of power and resources in the collection, analysis, and use of data related to the pandemic. 30 These power imbalances can occur at multiple levels, including within and between countries, and can have significant consequences for the effectiveness of COVID-19 responses and the distribution of benefits and harms. 30 Addressing these power imbalances is important for ensuring that data-driven interventions are effective and equitable, and for building trust and confidence in the use of data. This can involve efforts to increase the capacity and expertise of low- and middle-income countries (LMICs) to collect and analyze data, as well as efforts to ensure that data-driven interventions are transparent, inclusive, and accountable. 31 32

Decolonizing big data in global health, including in the context of COVID-19, involves acknowledging and addressing these power imbalances and working to create a more diverse and inclusive research environment. This can include collaborating with political leaders and advocacy groups to use big data to promote policies and practices that benefit marginalized communities, increasing the proportion of researchers from these communities, and incorporating traditional knowledge and practices into epidemiological research. It is also important to recognize and value the expertise of traditionally underserved populations, and to ensure that big data are used ethically and responsibly, with appropriate safeguards in place to protect the privacy and security of individuals. The COVID-19 pandemic has brought these issues to the forefront and has underscored the importance of decolonizing big data in global health to address health inequities. 29 A decolonized epidemiological data infrastructure should center on the knowledge of indigenous and marginalized populations and focus on intersectionality and interdependence. It should prioritize community-centered approaches, invest in diverse local leaders and scientists, and build algorithmic transparency and accountability. Consent from the population must be emphasized, ensuring that individuals have control over their data and its usage. To address the digital divide, facilitating access and communication for all members of the population is essential, and a clear and shared understanding of decolonization and data sovereignty is necessary to create an effective and equitable epidemiological data infrastructure.

In addition, the way that research has been conducted has often been shaped by the biases and assumptions of the researchers, leading to a lack of cultural sensitivity and a failure to consider the unique experiences and perspectives of different communities. 33 Another way in which colonial and imperialistic ideologies have shaped epidemiology is through the way that the results of research have been used. 34 35 36 In many cases, the results of epidemiological research have been used to justify policies and practices that disproportionately affect vulnerable communities. For example, epidemiological research has documented the harmful effects of lead exposure on cognitive development and other health outcomes, particularly in children. 37 38 39 However, policies and practices related to lead abatement and remediation have not always been applied equitably, and marginalized communities such as low-income and racial/ethnic minority populations may be disproportionately exposed to lead hazards. 3 40 This has contributed to a legacy of discrimination and injustice within the field, and it is essential that efforts are made to ensure that research is used in a more equitable and just manner.

Indigenous knowledge has been consistently undermined by colonialism, which elevates Eurocentric science as superior. 40 41 42 43 In the current COVID-19 pandemic response, epidemiology has not challenged this hierarchy of knowledge but rather reinforced it. 44 45 Epidemiology was rapidly and widely valued as a discipline and a group of experts without much questioning. This may be because the nation has long prioritized Westernized science since colonization, promoting the belief that it can save us from ourselves. Epidemiologists are seen as “expert knowers” and their science is viewed as unbiased, objective, and neutral. Even though in recent time many epidemiological researchers are careful not to conduct unethical studies to avoid sanctions, it is important to go beyond simply avoiding unethical practices and actively work to decolonize the field.

## Ethical and Respectful Research Practices

Ethical and respectful research practices are crucial in ensuring the decolonization of epidemiology and promoting inclusivity and equity. One of the fundamental aspects of ethical research is obtaining the full participation and informed consent of the communities being studied. To achieve this, researchers should work with local partners and community leaders to design and conduct research in a way that is sensitive to the needs and perspectives of the community. 46 Such efforts may involve engaging with the community to identify their priorities and concerns and involving them in the research process in a meaningful way. 47 Additionally, researchers should adapt research methods and protocols to be culturally appropriate and respectful of local customs and beliefs. 3 48 This may include modifying data collection methods, involving community members in research design, and adjusting interpretation and dissemination of findings. By providing concrete examples and fostering meaningful community engagement, researchers can conduct culturally sensitive and scientifically rigorous research.

Another essential aspect of ethical research is ensuring that the benefits of the research are shared with the community. 49 This means sharing the results of the research with the community in a way that is accessible and understandable and working with the community to ensure that the findings are used to address their needs and concerns. For instance, in a study conducted in rural Kenya, researchers worked with local partners and community leaders to design and conduct research on the impact of a new water treatment technology on child health. 50 The researchers engaged with the community to identify their needs and concerns and involved them in the research process, including training community members to collect data. The researchers also shared the results of the study with the community and worked with them to identify ways to use the findings to improve child health in the community. During the Ebola outbreak in West Africa from 2014 to 2016, Western researchers from various institutions and organizations worked closely with local communities and governments to understand and respond to the outbreak. 51 52 Such efforts included conducting epidemiological research to understand the spread and impact of the disease, working with local healthcare providers to develop and implement treatment and prevention strategies, and engaging with local communities to address their concerns and needs. These efforts required close collaboration and communication between Western researchers and local partners and were critical to the successful response to the outbreak.

Conducting epidemiological research in an ethical and respectful manner involves working with local partners and community leaders to design and conduct research that is sensitive to the needs and perspectives of the community and ensuring that the benefits of the research are shared with the community. 53 This approach fosters inclusivity, equity, and trust between researchers and the community, which are essential to decolonizing epidemiology.

## Diversity and Inclusion in Epidemiological Research

Another important step is to increase the diversity of the field itself. Epidemiology has traditionally been dominated by Western researchers, 54 55 56 and it is important to ensure that a wider range of voices and perspectives are represented within the field. This includes increasing the representation of researchers from marginalized communities, such as indigenous communities, people with disabilities, and communities of color among others. One way to increase the diversity of the field is by actively recruiting and supporting researchers from these communities and providing them with the resources and support they need to succeed. This could include providing mentorship and professional development opportunities, as well as offering financial support and other resources. It is also important to ensure that the research being conducted is relevant to the needs and experiences of marginalized communities. 57 This may involve working with these communities to identify their research priorities and ensuring that the research addresses these priorities. 58 For example, if a community is concerned about access to clean water, researchers could conduct studies on the impact of water quality on health outcomes and work with the community to identify solutions to improve access to clean water.

In addition to increasing the diversity of the field, it is also important to ensure that the results of epidemiological research are used in a more equitable and just manner. This may involve working with policymakers and advocacy groups to ensure that research is used to inform policies and practices that benefit marginalized communities, rather than being used to justify policies that disproportionately affect these communities. 59 For example, if research has shown that certain policies or practices disproportionately impact indigenous communities, researchers could work with indigenous advocacy groups and policymakers to identify alternative policies that would be more beneficial to these communities. This could involve advocating for policies that prioritize the rights and needs of indigenous communities, such as policies that protect traditional lands and resources. Although involving researchers in policy making is essential, it is crucial to address potential conflicts of interest or biases that may arise from their involvement. Ensuring transparency and objectivity in research will maintain the credibility and integrity of the research process.

## Power Imbalances in Epidemiological Research

Recognizing and addressing the ways in which power imbalances have shaped the way that health and disease have been understood and studied is an important aspect of decolonizing epidemiological research. Power imbalances, such as those based on colonialism, imperialism, and globalization (political factors), have had a significant impact on the way that health and disease have been understood and studied. 60 61 62 One way in which power imbalances have shaped the way that health and disease have been understood and studied is through the allocation of research funding. 63 64 For example, many diseases that are considered to be major health problems in the global North (such as heart disease and cancer) may receive a disproportionate amount of research funding and attention, while diseases that disproportionately affect marginalized communities (such as infectious diseases and neglected tropical diseases) may receive far less attention. This can lead to a lack of understanding and effective interventions for these diseases, and it is important to ensure that research funding and attention are more equitably distributed. In the context of the COVID-19 pandemic, power imbalances have been particularly evident in the distribution of resources and attention. 65 For example, wealthier countries and research institutions have often had greater access to funding and resources, which has enabled them to conduct more extensive research on the virus and its impacts. This has led to concerns about the potential for global health inequities, as the needs and experiences of poorer countries and communities may not be adequately represented in the research being conducted. 66

Another way in which power imbalances have shaped the way that health and disease have been understood and studied is through the way that research is conducted, and knowledge is produced. 67 For example, many early studies on health and disease were conducted by Western researchers in colonized countries, and this has led to a focus on Western perspectives and a lack of attention to the experiences and perspectives of marginalized communities. 68 In some cases, power imbalances can manifest in the way that research questions are framed and studied. 69 For example, if research questions are driven by the interests of those in positions of power, the needs and experiences of marginalized communities may be overlooked or ignored. 69 This can result in research that does not accurately represent the experiences of those most affected by a particular health issue. It is important to ensure that research is conducted to promote the inclusion of diverse perspectives in research and decision-making. 70 Overall, recognizing and addressing the ways in which power imbalances have shaped the way that health and disease have been understood and studied is an important step in decolonizing epidemiological research and creating a more inclusive and equitable approach. This involves ensuring that research funding and attention are more equitably distributed and promoting the inclusion of diverse perspectives in research and decision-making.

## Social and Economic Factors in Epidemiological Research

Recognizing that the social and economic factors that contribute to health and disease are often shaped by colonialism and other forms of oppression 71 is an important aspect of decolonizing epidemiological research. It is important to acknowledge and address these underlying factors, rather than simply focusing on individual behaviors or risk factors, to create a more inclusive and equitable approach. One example of how colonialism has shaped the social and economic factors that contribute to health and disease is in indigenous communities. 72 Indigenous communities may experience higher rates of certain diseases, 72 such as diabetes and heart disease, due to the impact of colonization on their traditional ways of life and access to healthcare. For example, the forced removal of indigenous communities from their traditional lands and the disruption of their traditional practices and ways of life may contribute to the development of certain health conditions. 73 Additionally, indigenous communities may have limited access to healthcare due to discrimination and other barriers, such as geographic isolation and lack of transportation. 74 75 Another example is the way that structural racism and discrimination contribute to health disparities in communities of color. 76 Communities of color may experience higher rates of certain diseases, such as hypertension and obesity, due to factors such as lower income and education levels, lack of access to healthy food options, and exposure to environmental toxins. 76 Structural racism and discrimination may also impact access to healthcare and contribute to poorer health outcomes. 77

Decolonizing epidemiological research involves acknowledging and addressing these underlying social and economic factors, rather than simply focusing on individual behaviors or risk factors. This may involve working with communities to identify the root causes of health disparities and addressing these causes in a holistic and culturally appropriate manner. It may also involve advocating for policies and practices that address structural inequalities and promote health equity. For example, to address the higher rates of diabetes and heart disease in indigenous communities, researchers could work with these communities to identify the root causes of these health disparities and develop interventions that address these causes in a culturally appropriate manner. This could involve working with the community to promote traditional practices and ways of life, such as traditional diets and physical activity, as well as addressing barriers to healthcare access.

To address the health disparities experienced by communities of color, researchers could work with these communities to identify the root causes of these disparities and develop interventions that address these causes. This could involve advocating for policies and practices that address structural inequalities, such as addressing environmental toxins and promoting access to healthy food options, as well as addressing barriers to healthcare access. It is generally accepted that investment in social epidemiology is important in the modern world. 78 79 Social epidemiology is a field that focuses on the social and economic factors that contribute to health and disease in populations. 80 These factors include things like income, education, housing, and access to healthcare, and they are often shaped by structural inequalities such as racism, discrimination, and colonialism. 81 By studying these factors, social epidemiologists aim to understand the root causes of health disparities and develop interventions to address them. Given the ongoing challenges of health inequities and social injustice, many experts believe that increasing investment in social epidemiology is necessary to address these issues and promote health equity. 79 80 81 Overall, recognizing and addressing the social and economic factors that contribute to health and disease, and the ways in which these factors are shaped by colonialism and other forms of oppression, is an important step in decolonizing epidemiological research and creating a more inclusive and equitable approach.

## Authorship in Epidemiological Research and Journal Editorship

Decolonizing authorship in epidemiological research refers to efforts to ensure that research is conducted and led by a diverse group of researchers, including those from traditionally underserved or marginalized communities. This is an important issue, as power imbalances and inequities in authorship in epidemiological research have been a longstanding concern, 82 83 84 with research often being led and conducted by researchers from dominant or privileged groups, while the contributions of researchers from marginalized or underserved communities have been underrepresented or unrecognized. This disparity is evident in a study of 882 papers involving 10,570 authors across 61 LMICs. Compared with authors with high-income country (HIC)-only affiliations, authors with LMIC-only affiliations were less likely to hold first or last authorship positions, while those with mixed HIC/LMIC affiliations had greater likelihood. 84 Furthermore, the proportion of senior authors with LMIC-only affiliations was lowest in the highest impact journals and in multicountry studies compared with single-country studies, highlighting the ongoing challenges in addressing inequities within the research landscape. 84

Decolonizing authorship in epidemiological research can help to create a more inclusive and equitable field and can also have several other benefits. One reason why decolonizing authorship in epidemiological research is important is that it can help to ensure that research is more relevant and meaningful to a wider range of communities. Researchers from marginalized or underserved communities may have unique insights and perspectives that can inform the design and conduct of research and can help to ensure that research addresses the needs and concerns of these communities. 85 Involving researchers from these communities can also help to build trust and confidence in the research among local communities, which can be critical for the success of research studies and for the uptake of research findings. 85 Another reason why decolonizing authorship in epidemiological research is important is that it can help to increase the impact and influence of research. Research that is conducted and led by a diverse group of researchers is more likely to be representative of the populations it seeks to study and is more likely to be relevant and applicable to a wider range of contexts. 86 87 This can help to increase the generalizability and usefulness of research findings and can also increase the likelihood that research findings will be used to inform policies and practices that benefit marginalized or underserved communities.

There are several ways in which authorship in epidemiological research can be decolonized. One important strategy is to promote diversity and inclusion in research training programs and career development opportunities. 88 This can involve providing financial and other forms of support to researchers from marginalized or underserved communities, as well as creating opportunities for these researchers to gain experience and build their skills and networks. It can also involve creating more inclusive and supportive research environments and working to address structural barriers and biases that may prevent researchers from these communities from participating fully in the research process. Researchers from marginalized communities face barriers and biases such as limited access to education, resources, funding, and networking opportunities, as well as implicit discrimination and underrepresentation in decision-making processes. These challenges hinder their ability to fully participate in and contribute to epidemiological research, perpetuating disparities in authorship. Another important strategy is to support the development of research capacity in marginalized or underserved communities. This can involve providing resources and support to help these communities conduct their own research, as well as building partnerships and collaborations between researchers from these communities and researchers from more privileged groups. It can also involve working with local organizations and institutions to help them develop the skills and resources they need to conduct and use research effectively.

While decolonizing authorship in epidemiological research has many benefits, it is crucial to acknowledge potential limitations and challenges. Some concerns include tokenism, where researchers from marginalized communities may be included solely to meet diversity requirements, rather than genuinely valuing their perspectives and contributions. 89 Additionally, promoting diversity may initially slow down research processes due to the need for additional resources and time to ensure proper representation and collaboration. Academic institutions should be aware of these potential issues and implement measures to address them. For instance, they could establish guidelines and protocols to ensure genuine engagement with diverse researchers and communities, rather than mere tokenism. 86 Institutions should also allocate sufficient resources and time to support meaningful collaborations, recognizing that the long-term benefits of more inclusive research outweigh the initial investment.

In addition to the ongoing discussion surrounding the decolonization of authorship in epidemiological research, there is a growing emphasis on the need to decolonize editorship in epidemiological journals and other global health journals. 90 91 In response to this, a study presents a new scoring system called the Composite Editorial Board Diversity Score (CEBDS) to evaluate the diversity of editorial boards in terms of three parameters—gender, country income-level, and geographic region. 92 The diversity of the editorial boards of 27 specialty global health journals was analyzed, revealing that of 303 editors, 40% were females, 68% were based in HICs, 34% in Europe and Central Asia, and 30% in North America. Among editors-in-chief, 27% were females and 73% were based in HICs. Only 26% of journals achieved the highest possible score in the gender diversity domain (40–60% female editors), 11% in the country income-level domain (at least one editor in all country income groups), and 7% in the geographic region diversity domain (at least one editor in all six regions). Overall, a mere 11% of journals had high CEBDS (≥8). Further studies are needed to understand the enablers and barriers of diversity in journal editorial boards, and affirmative action and organizational good practices for improving diversity, inclusion, and belongingness must be implemented to ensure diversity in the editorial boards of epidemiology and global health journals.

## Traditional Knowledge and Practice in Epidemiological Research

Part of the effort to decolonize epidemiological research must emphasizes the unique role of both qualitative and quantitative research as key components in the field. By recognizing and valuing the contributions of diverse methodological approaches, researchers can develop a more inclusive, equitable, and comprehensive understanding of public health issues and health inequities. 20 93 94 While mixed-methods research combines qualitative and quantitative approaches and can contribute to a more comprehensive understanding of public health issues, it was not initially set up explicitly to decolonize epidemiological research. However, embracing mixed-methods research can be an important step in the decolonization process, as it fosters a more inclusive and equitable research environment by valuing diverse perspectives and methodologies. This approach can help dismantle the hierarchies that have been established in the field and promote research practices that better serve the needs of marginalized and underserved communities. This shift in perspective can help promote more culturally sensitive research practices that respect and involve the perspectives and needs of marginalized and underserved communities. In the context of local communities, researchers may face the dilemma of balancing respect for traditional values while addressing practices that negatively impact public and personal health. Navigating this challenge requires engaging in open and respectful dialogue with community members, acknowledging their expertise, and collaboratively developing culturally sensitive interventions. This approach promotes mutual understanding and encourages the adoption of healthier practices while respecting cultural values and beliefs.

Many indigenous communities have a rich history of traditional medicine and healing practices, 95 96 and it is important to recognize and incorporate this knowledge into research and healthcare practice. This involves acknowledging the value of traditional knowledge and practices and working with traditional healers and community leaders to design and conduct research. Traditional knowledge and practices refer to the cultural, historical, and experiential knowledge and practices that are passed down through generations within a particular community or culture. 97 This includes knowledge and practices related to health, wellness, and the natural environment, as well as social and cultural practices and traditions.

Incorporating traditional knowledge and practices into epidemiological research offers a multitude of benefits, particularly in indigenous or marginalized communities where such knowledge plays a pivotal role in health and well-being. As global health expert Dr. Seye Abimbola points out, there has been an ongoing epistemic injustice that distances us from the valuable knowledge found at the periphery, often due to colonial legacies. 98 By integrating traditional knowledge and practices into research, we can ensure that it is not only relevant and meaningful to local communities but also takes into account their cultural and historical context. This respectful and responsive approach to research is more likely to be accepted and utilized by local communities, increasing the uptake and effectiveness of research findings. Furthermore, acknowledging, and valuing knowledge from the periphery helps to repair the damage caused by colonialism and promotes mutual understanding and respect between researchers and local communities. By working together and sharing knowledge and expertise, both parties can learn from each other, building stronger partnerships and collaborations, and ultimately challenging the colonial conceit that has perpetuated epistemic injustice.

## Conclusion

It is important to recognize that decolonizing epidemiological research is not a one-time process, but rather an ongoing journey. Decolonizing epidemiological research is a crucial step in creating a more just and equitable world and involves acknowledging and addressing the ways in which colonial and imperialistic ideologies have shaped the field and working to increase the diversity and inclusivity of the field. It also involves recognizing and addressing the underlying social and economic factors that contribute to health and disease and valuing the knowledge and expertise of marginalized communities. By taking these steps, we can ensure that epidemiology is used to benefit all members of society, rather than being used to further the agendas of the dominant power structures.
